# Comparison of the effects of the three major tyrosine kinase inhibitors as first-line therapy for non-small-cell lung cancer harboring epidermal growth factor receptor mutations

**DOI:** 10.18632/oncotarget.24386

**Published:** 2018-02-04

**Authors:** Chih-Yen Tu, Chuan-Mu Chen, Wei-Chih Liao, Biing-Ru Wu, Chih-Yu Chen, Wei-Chun Chen, Te-Chun Hsia, Wen-Chien Cheng, Chia-Hung Chen

**Affiliations:** ^1^ Department of Life Sciences, and Agricultural Biotechnology Center, National Chung Hsing University, Taichung, Taiwan; ^2^ Division of Pulmonary and Critical Care Medicine, Department of Internal Medicine, China Medical University Hospital, Taichung, Taiwan; ^3^ School of Medicine, China Medical University, Taichung, Taiwan; ^4^ Department of Respiratory Therapy, China Medical University, Taichung, Taiwan; ^5^ Graduate Institute of Clinical Medical Science, China Medical University, Taichung, Taiwan; ^6^ Department of Internal Medicine, Hyperbaric Oxygen Therapy Center, China Medical University, Taichung, Taiwan; ^7^ Taiwan Clinical Trial Consortium for Lung Diseases (TCoC), Taichung, Taiwan

**Keywords:** EGFR-mutated lung adenocarcinoma, gefitinib, erlotinib, afatinib, progression-free survival

## Abstract

**Introduction:**

Patients with advanced lung adenocarcinoma harboring epidermal growth factor receptor (EGFR)-activating mutations have good response rate and longer progression-free survival (PFS) when treated with the tyrosine kinase inhibitors (TKI) compared with platinum-based chemotherapy. However, studies comparing the effectiveness of these drugs as first-line therapy in such patients are limited.

**Results:**

We analyzed 422 patients with EGFR-mutated advanced lung adenocarcinoma receiving first-line gefitinib (*n* = 195, 46.2%), erlotinib (*n* = 123, 29.1%), or afatinib (*n* = 104, 24.6%). The PFS of the afatinib group was longer (12.2 months) than that of the gefitinib group (9.8 months) (*p* = 0.035) but similar to that of the erlotinib group (11.4 months) (*p* = 0.38). In patients without brain metastasis (BM), subgroup analysis showed that the afatinib group had significantly longer PFS (13.1 months) than erlotinib (11.7 months) and gefitinib (9.8 months) groups (*p* = 0.010). Patients with exon 19 deletions in the afatinib and erlotinib groups had potentially long PFS (*p* = 0.073). Efficacy of afatinib was similar between the 30 mg and 40 mg arms (median PFS 16.1 months vs. 10.3 months; *p* = 0.923).

**Conclusions:**

Afatinib may be the optimal EGFR-TKI for advanced lung adenocarcinoma harboring EGFR-activating mutations, particularly in the absence of BM. Patients with exon 19 deletions taking afatinib had potentially long PFS. An afatinib dose of 30 and 40 mg has similar effect.

**Methods:**

We conducted this retrospective study at a single medical center from January 2013 to March 2017 and used PFS to evaluate the effectiveness of gefitinib, erlotinib, and afatinib in patients with advanced lung adenocarcinoma harboring EGFR mutations.

## INTRODUCTION

Advanced non-small-cell lung cancer (NSCLC) that harbors characteristic mutations in epidermal growth factor receptor (EGFR) is highly sensitive to EGFR tyrosine kinase inhibitors (TKIs) [1]. The frequency of EGFR mutations varies widely across different populations, with increased incidence of such mutations in nonsmokers [2], women [3], adenocarcinomas [4, 5], and the Asian population [6]. The incidence of EGFR mutations was approximately 50% and 10–15% among Asian and Caucasian NSCLC patients, respectively [7].

Three major EGFR-TKIs, namely, gefitinib, erlotinib, and afatinib, have been approved for NSCLC with EGFR mutations since 2009. Gefitinib and erlotinib are the first-generation EGFR-TKIs that reversibly inhibit the kinase activity of overall EGFR (HER1). Afatinib, a second-generation EGFR-TKI, covalently and irreversibly binds to the intracellular tyrosine kinase domain and is a highly selective blocker of the pan-ErbB family [8]. It inhibits intracellular phosphorylation, preventing further downstream signaling and resulting in cell death. The three major EGFR-TKIs had demonstrated superior response rate (RR) and significantly prolonged progression-free survival (PFS) but not in overall survival (OS) in phase III trial in patients with NSCLC harboring EGFR mutations compared with platinum-based chemotherapy doublets [4, 5, 9–16]. Interestingly, the combined analyses of LUX-Lung 3 and LUX-Lung 6 indicated that afatinib had a statistically significant benefit in OS in patients with exon 19 deletion [17]. Interest on which EGFR-TKI should be the best choice as first-line therapy in such patients has been growing.

Few clinical trials conducted a head-to-head comparison of EGFR-TKIs. Two phase III trials that directly compared erlotinib and gefitinib were conducted in Asian patients; these two agents are comparably effective in previously treated EGFR mutation-positive NSCLC patients [18, 19]. In the phase IIb LUX-Lung 7 trial comparing gefitinib and afatinib as first-line treatment of EGFR-mutated NSCLC patients, afatinib significantly improved PFS compared with gefitinib (11.0 months vs. 10.9 months; HR: 0.73; *p* = 0.017) [20], and no significant difference in OS was noted in a subsequent report [21].

To date, no trial has compared these three TKIs together. A limited number of retrospective studies compared these three TKIs. Kuan *et al*. reported that PFS was significantly longer in patients who received afatinib and erlotinib compared with those who received gefitinib as first-line treatment of common EGFR-mutated NSCLC [22]. Meanwhile, Krawczyk *et al*. reported that the effectiveness (treatment response, median PFS, and OS) of these three TKIs was not significantly different in patients with both common and rare EGFR mutations [23]. Therefore, we conducted a retrospective study to analyze the effectiveness of these three EGFRTKIs as first-line therapy in NSCLC patients with EGFR mutations.

## RESULTS

### Patient characteristics

**Figure 1:**
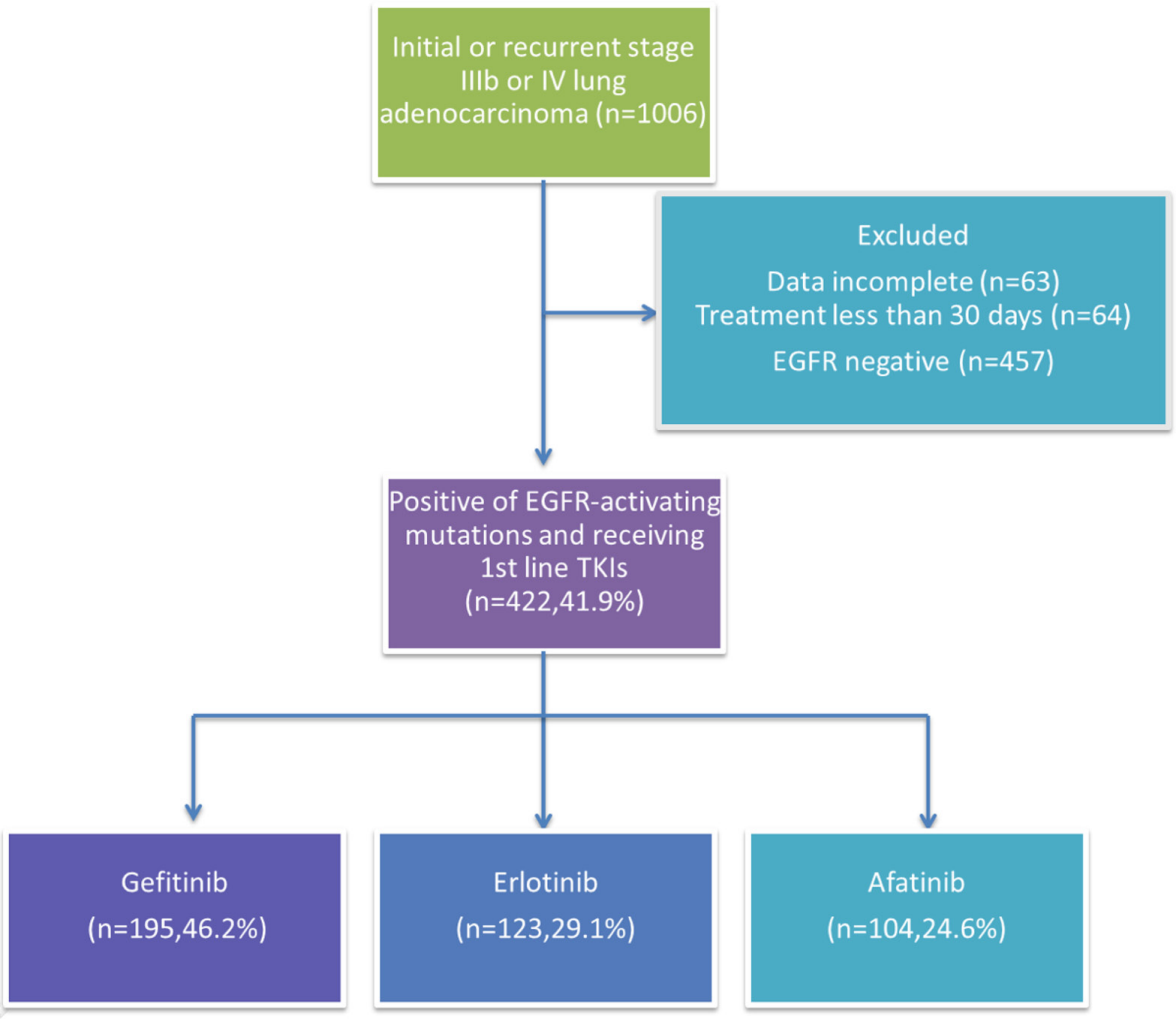
Patient disposition

**Figure 2:**
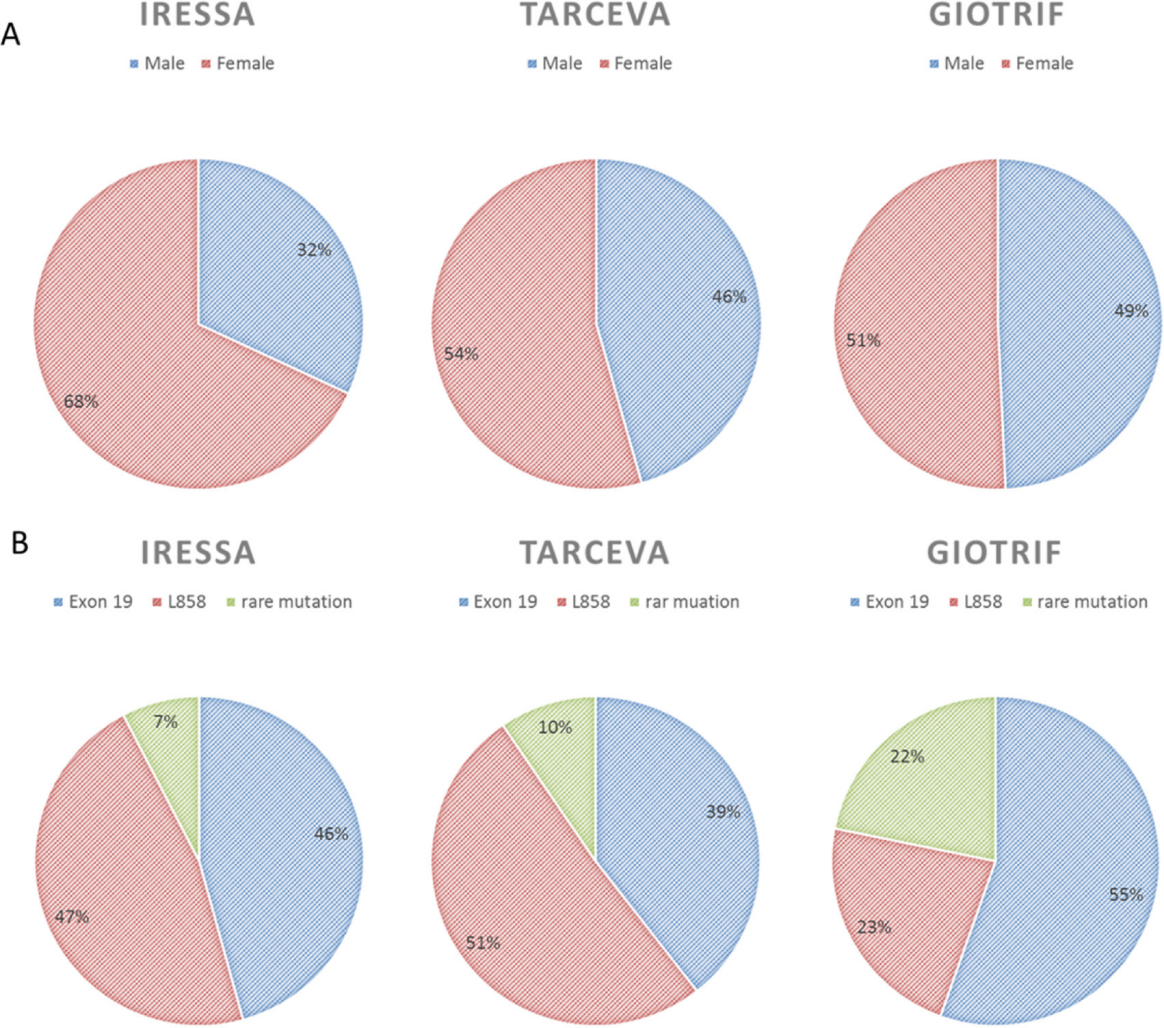
The three EGFR-TKIs proportions by (**A**) gender (**B**) the type of EGFR mutations.

A total 1951 patients were screened between January 2013 and March 2017, 1006 of whom had newly diagnosed or recurrent stage IIIb/IV lung adenocarcinoma. Among them, 457 patients had tumors that were EGFR mutation negative (wild-type EGFR). A total of 63 patients were excluded from the study because of incomplete data, and 64 were excluded in the analysis their treatment lasted less than 30 days. Four hundred and twenty-two patients with EGFR mutation-positive advanced lung adenocarcinoma received gefitinib (*n* = 195), erlotinib (*n* = 123), or afatinib (*n* = 104) as first-line treatment (Figure 1). Baseline characteristics of the patients are shown in Table 1. Significant differences were noted in gender (*p* = 0.043) and age (*p* = 0.044), while the other factors were not statistically significant between the treatment groups. The proportion of elderly patients (56.9%) and women (69.7%) was higher in the gefitinib group than in the other two groups (Figure 2A). However, the result showed a slight difference in the composite of the types of EGFR mutation in each arm (*p* = 0.058). The afatinib group had a high percentage of exon 19 deletions (55.8%) and rare mutation (22.1%) and a low percentage of Leu858Arg (22.1%) (Figure 2B). We performed Cox regression analysis to adjust the variations.

**Table 1:** Baseline characteristics of NSCLC patients according to EGFR-TKIs

	Gefitinib	Erlotinib	Afatinib	*P* value
	*N* = 195	*N* = 123	*N* = 104	
Sex				0.043
Men	59 (30.3)	54 (43.9)	39 (37.5)	
Women	136 (69.7)	69 (56.1)	65 (62.5)	
Age (years)				0.044
<65	84 (43.1)	68 (55.3)	58 (55.8)	
>65	111 (56.9)	55 (44.7)	46 (44.2)	
Smoking				0.446
Never	147 (75.4)	92 (74.8)	86 (82.7)	
Current or ever	48 (24.6)	31 (25.2)	18 (17.3)	
BMI				0.713
<20	27 (13.8)	21 (17.1)	17 (16.3)	
>20	168 (86.2)	102 (82.9)	87 (83.7)	
EGFR mutation				0.058
Del19	87 (44.6)	48 (39)	58 (55.8)	
L858R	94 (48.2)	63 (51.2)	23 (22.1)	
Clinical stage				0.543
IIIb	9 (4.6)	3 (2.4)	3 (2.9)	
IV	186 (95.4)	120 (97.6)	101 (97.1)	
ECOG PS				0.332
0 & 1	164 (84.1)	109 (88.6)	93 (89.4)	
> 1	31 (15.9)	14 (11.4)	11 (10.6)	
Baseline brain metastasis				0.360
Absence	161 (82.6)	105 (86.1)	82 (78.8)	
Presence	34 (17.4)	17 (13.9)	22 (21.2)	

### Progression-free survival

**Figure 3:**
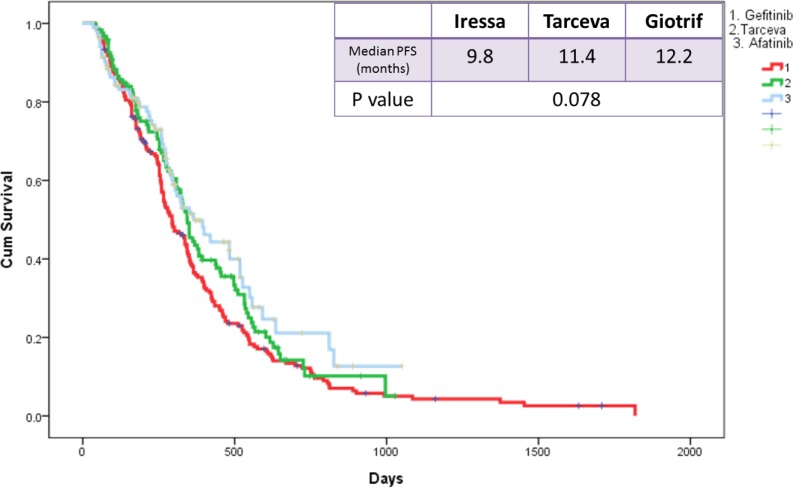
Kaplan-Meier survival curves of progression-free survival in patients received gefitinib, erlotinib, and afatinib

**Figure 4:**
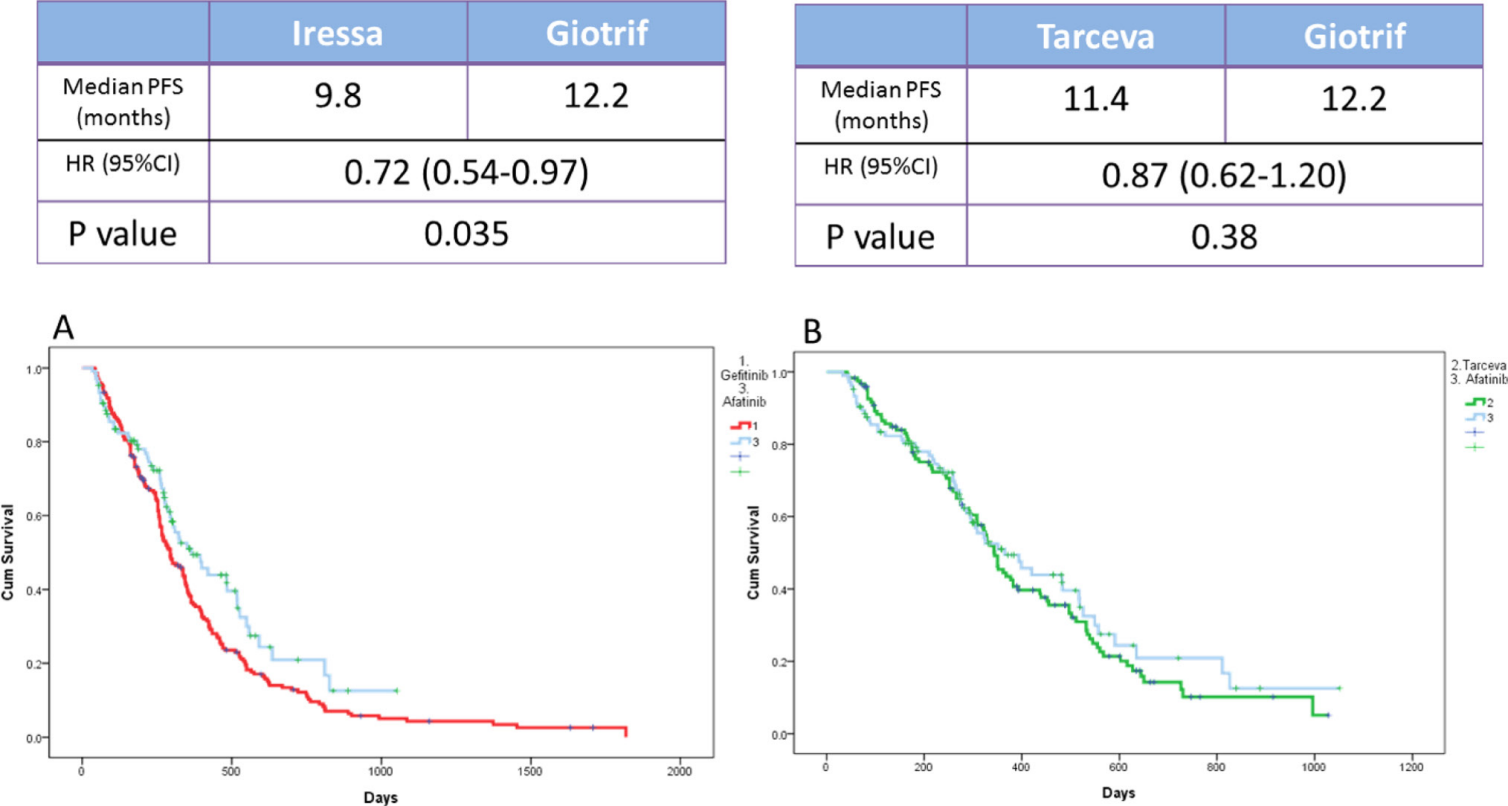
Kaplan-Meier survival curves of progression-free survival in patients who received (**A**) gefitinib and afatinib and (**B**) erlotinib and afatinib.

**Figure 5:**
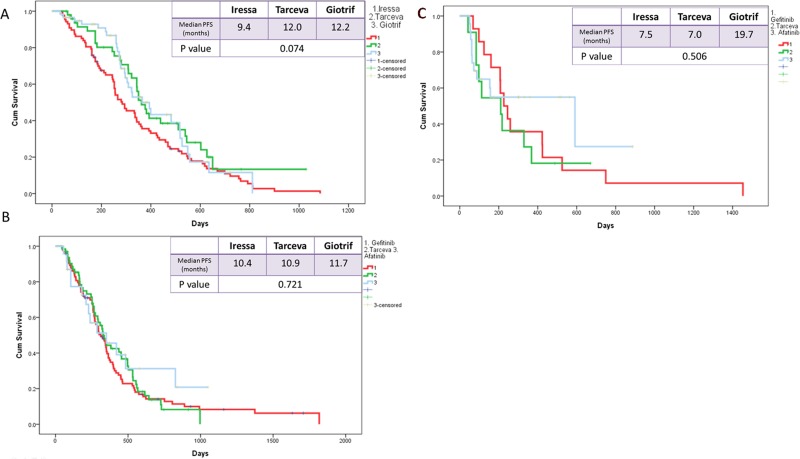
Kaplan-Meier survival curves of progression-free survival in patients who received gefitinib, erlotinib, and afatinib (**A**) in exon 19 deletions (**B**) in Leu858Arg (**C**) rare mutations.

The median PFS of the three EGFR TKI patient groups (gefitinib, erlotinib, and afatinib) was 9.8, 11.4, and 12.2 months, respectively (Figure 3). Patients receiving afatinib had a significantly longer PFS than did patients receiving gefitinib (median, 12.2 vs. 9.8 months; *p* = 0.035; Figure 4A) but had similar PFS with those receiving erlotinib (median, 12.2 vs. 11.4 months; *p* = 0.38; Figure 4B) in the entire study population. Analysis results based on the type of EGFR mutations showed that PFS was not significantly different among the three EGFR TKIs. However, in patients with exon 19 deletions, the afatinib or erlotinib group had slightly longer PFS than the gefitinib group (12.2 vs. 12.0 vs. 9.4 months; *p* = 0.074; Figure 5A, 5B). In patients with rare EGFR mutation, the afatinib group (19.7 months) had longer PFS than the erlotinib (7.0 months) and gefitinib (7.0 months) groups, although the difference was not statistically significant (19.7 months vs. 7.0 months vs. 7.0 months, respectively; *p* = 0.506; Figure 5C).

**Figure 6:**
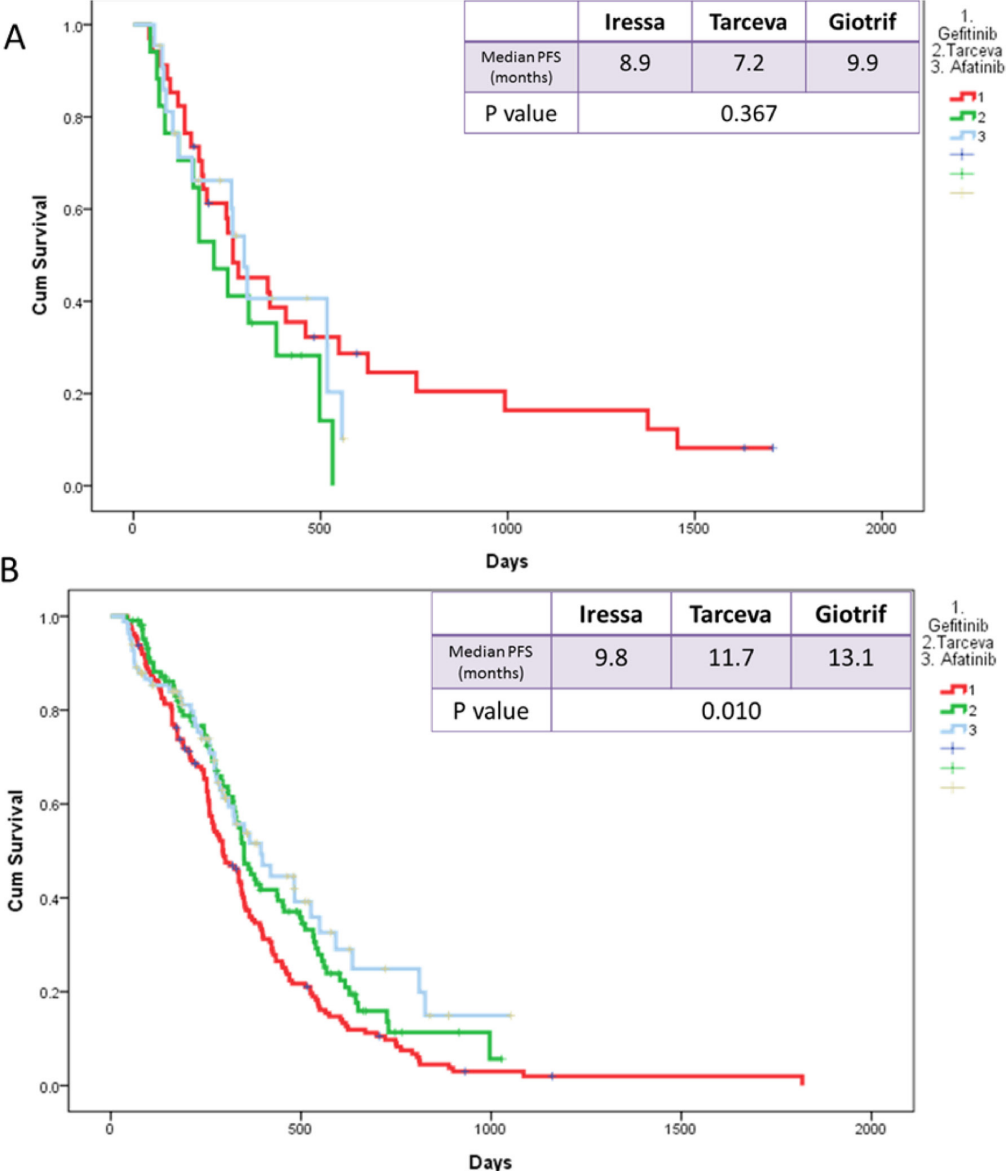
Kaplan-Meier survival curves of progression-free survival in patients who received gefitinib, erlotinib, and afatinib (**A**) in brain metastasis and (**B**) in no brain metastasis.

PFS was also not significantly different among in subgroups that were based on such factors as gender (*p* = 0.404 for male and *p* = 0.078 for female), smoking status (*p* = 0.12 for smokers and *p* = 0.148 for nonsmokers), and presence of brain metastasis (BM) (*p* = 0.376; Figure 6A). However, in the subgroup with no BM, afatinib was associated with significantly longer median PFS than erlotinib or gefitinib (13.1 months, 11.7 months, and 9.8 months, respectively; *p* = 0.010; Figure 6B).

**Figure 7:**
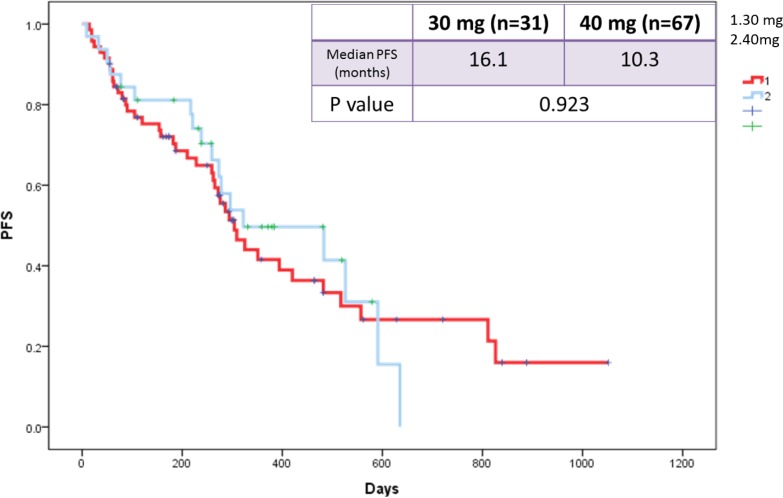
Kaplan-Meier survival curves of progression-free survival in patients who received afatinib doses of 30 mg and 40 mg

We also evaluated the influence of afatinib dose reduction on PFS. The median PFS was compared in patients in whom afatinib dose was reduced to 30 mg vs. those whose doses were maintained at 40 mg. The results indicated that the median PFS was similar in patients in whom the dose was reduced to 30 mg (16.1 months) vs. those remaining at 40 mg (10.3 months) (*p* = 0.923; Figure 7).

## DISCUSSION

Our study showed that in NSCLC patients with EGFR mutations, afatinib was superior to gefitinib but had similar effectiveness to erlotinib. In the patient subgroup with no BM, afatinib provided significantly longer PFS than erlotinib or gefitinib. Patients with exon 19 deletions and rare mutations treated with afatinib had slightly longer PFS than those receiving the first-generation TKIs. Moreover, the effectiveness of afatinib was similar between the doses 30 mg and 40 mg.

The result of the current study was consistent with the findings of LUX-Lung 7 trial. Afatinib significantly improved PFS (11.0 month and 10.9 months; HR: 0.73; *p* = 0.017) and time to treatment failure (13.7 months and 11.5 months; HR: 0.73; *p* = 0.007) compared with gefitinib. [20] Our study even indicated that afatinib was associated with significantly longer PFS compared with erlotinib or gefitinib (13.1, 11.7, and 9.8 months, respectively; *p* = 0.010) in the analysis of the patient subgroup with no BM. Such result may be explained by the different mechanisms of action between first-generation and second-generation EGFR-TKIs. The first-generation EGFR-TKIs reversibly bind to and inhibit EGFR signaling, while the second-generation EGFR-TKIs irreversibly binds to and blocks signaling from the homo- or heterodimers of pan-ErbB family receptors [8].

EGFR-TKIs for the treatment of brain metastases (BM) in patients with EGFR mutation-positive NSCLC has been receiving increasing attention. A phase II study indicated a favorable response of BM to gefitinib. The response rate (RR), median PFS, and median OS for BM were 87.8%, 14.5 months, and 21.9 months, respectively [24]. A retrospective study showed that erlotinib is effective in BM from NSCLC with EGFR activating mutations in exons 19 or 21. The RR for BM, the median time to progression in the brain, and median OS were 82.4%, 11.7 months, and 12.9 months, respectively [25]. The LUX-Lung 3 and 6 trial also showed the superiority of afatinib over chemotherapy in patients with EGFR mutation-positive NSCLC and BM (11.1 vs. 5.4 months; HR: 0.54; *p* = 0.1378) and (8.2 vs. 4.7 months; HR: 0.47; *p* = 0.1060) [26]. Head-to-head comparisons among gefitinib, erlotinib, and afatinib for patients with BM are yet to be conducted. In the present study, no significant difference was noted in PFS in patients treated with these drugs. The median PFS of patients treated with gefitinib, erlotinib, and afatinib was 8.9, 7.2, and 9.9 months, respectively (*p* = 0.367). The median PFS in our results was shorter than that in previous studies, which may be due to a high percentage of rare EGFR mutations in our study (gefitinib: 7%, erlotinib: 7%, and afatinib: 21%).

The types of EGFR mutation may influence the effectiveness of EGFR-TKIs; patients with exon 19 deletions treated with gefitinib and erlotinib had longer survival than did patients with L858R mutation treated with the same medications [27–29]. A meta-analysis of 13 studies showed that exon 19 deletions might be associated with longer PFS compared with L858 mutations [30]. From this meta-analysis, afatinib showed higher efficacy in patients harboring exon 19 deletion than those with L858R mutation (HR: 0.49; *p* = 0.108) compared with gefitinib (HR: 0.76; *p* = 0.244) and erlotinib (HR: 0.53; *p* = 0.264). The results of the pooled LUX-Lung 3 and 6 analysis showed that afatinib had a statistically significant benefit for OS in patients with exon 19 deletions compared with standard chemotherapy [17]. Our study showed that afatinib and erlotinib had a similar trend of longer PFS than gefitinib in patients with exon 19 deletion (12.2, 12.0, and 9.4 months, respectively; *p* = 0.074), but no difference in patients with L858R mutation (11.7, 10.9, and 10.4 months, respectively; *p* = 0.721). These difference may be explained by the following reasons: (1) T790M mutation, which is associated with primary and acquired TKI resistance, might occur more frequently for L858R, and L858R can coexist more frequently with other rare EGFR mutations, affecting the EGFR kinase sensitivity to TKIs; (2) exon 19 deletion might be more actively inhibited by EGFR TKIs because of an increased affinity for these than L858R mutations [30].

The present study also showed that afatinib (19.7 months) causes potentially longer PFS than erlotinib (7.0 months) and gefitinib (7.0 months) in patients with rare EGFR mutations, although no statistically significant difference was noted, which may be due to the less number of patients in this study. Chiu *et al*. reported that the median PFS was 7.7 months in patients with rare mutations (G719X/L861Q/S768I) after first-generation EGFR-TKI treatment [31]. Yang *et al*. indicated that the median PFS of patients harboring these rare mutations (G719X/L861Q/S768I) treated with afatinib was 10.7 months [32]. According to these two studies, afatinib may be a first-choice EGFR-TKI for patients with rare EGFR mutations, particularly G719X, L861Q, and S768I.

The most common adverse events (AEs) of these three major EGFR-TKIs include skin rash, stomatitis, paronychia, and diarrhea, which are manageable with treatment interruptions or dose reduction and best supportive care [33]. We assessed the effectiveness of tolerability-reduced afatinib dose, and the median PFS was found to be similar between patients in whom the dose was reduced to 30 mg (16.1 months) and those remaining at 40 mg (10.3 months) (*p* = 0.923). The result was consistent with that of the LUX-Lung 3 and 6 studies (LL3: 11.3 vs. 11.0 months; HR: 1.25 and LL6: 12.3 vs. 11.0 months; HR: 1.00) [34]. These results indicate that dose adjustment does not only has no impact on therapeutic efficacy but also reduces afatinib-related AEs.

We acknowledge some limitations in our study. First, this was a retrospective analysis, and some bias may be present in our study. Second, the numbers of patients with rare EGFR mutation varied among the three arms, and the sample size of each arm was small, which may *result in no statistical significance*. Third, we did not identify the type of rare mutations; the effectiveness of EGFR-TKIs is highly variable depending on the mutation. G719X, 19 insertions, S768I, and L861Q may be sensitizing mutations [31, 32, 35] and *de-novo* Thr790Met and exon 20 insertion mutations are resistant mutations [36, 37]. Finally, we only used PFS to evaluate the efficacy of these three major EGFR-TKIs. We did not analyze the OS and the AEs of each arm. OS can be influenced by several factors, and skin rash and grade ≥3 diarrhea were more frequent with afatinib, while hepatotoxicity was more frequent with gefitinib [38]. PFS was evaluated dependent on our real-world practice with a tolerable dose of these drugs.

Afatinib may be the optimal EGFR-TKI in patients with advanced lung adenocarcinoma harboring EGFR-activating mutation, particularly in the absence of BM. Afatinib afforded potentially longer PFS in patients with exon 19 deletions and rare EGFR mutations. Reducing the afatinib dose to 30 mg does not affect its efficacy or the patient PFS. However, future prospective studies are warranted to determine the most appropriate EGFR-TKI for patients with NSCLC harboring EGFR mutations.

## PATIENTS AND METHODS

The study was performed retrospectively between January 2013 and March 2017 at the department of the Division of Pulmonary and Critical Care Medicine, China Medical University Hospital, which is a 2,146-bed community-based university hospital in Taichung, Taiwan. The study was approved by the China Medical University Hospital Internal Review Board (CMUH103-REC1-112), and written informed consent was obtained from all patients.

### Enrolled patients and clinical data

The patients inclusion criteria were as follows: (1) age > 18 years, (2) initial or recurrent stage IIIb or IV lung adenocarcinoma (as classified according to the American Joint Committee on Cancer AJCC TNM staging system, 7th edition) that had been diagnosed at CMUH between January 2013 and March 2017, (3) positive for EGFR mutation, and (4) received first-line EGFR-TKI (gefitinib, erlotinib, or afatinib). All relevant patient data were collected, including age, sex, smoking history, body mass index, types of EGFR mutations, types of EGFR-TKIs, clinical stage, and brain scan image.

### Diagnosis and treatment

Lung cancer was diagnosed via bronchoscopy, computed tomography (CT)-guided or ultrasound-guided lung biopsy, surgery, and malignant pleural effusion cytology. EGFR mutation was analyzed in patients diagnosed with advanced NSCLC. DNA was extracted from formalin-fixed tumor tissue or tumor cells obtained during the diagnostic or therapeutic procedure. EGFR gene mutations were tested via direct sequencing with routine realtime polymerase chain reaction procedures or the amplification refractory mutation system [39]. In Taiwan, three EGFR-TKIs, namely, gefitinib, erlotinib, and afatinib, are reimbursed by the National Health Insurance program as first-line treatment of patients with EGFR mutation-positive NSCLC.

EGFRTKIs were administered orally at a daily dose of 250 mg for gefitinib, 150 mg for erlotinib, and 40 mg for afatinib. Skin rash, stomatitis, paronychia, and diarrhea were common side effects during therapy. If intolerable treatment toxicity occurred, we reduced the amount of EGFRTKIs to the recommended dose. The treatment toxicity was assessed using the Common Toxicity Criteria scale (version 4.0). Treatment was continued until unacceptable toxicity or disease progression, and the patients received chemotherapy or palliative radiotherapy after stopping EGFR-TKIs.

### Assessments

PFS was defined as the date of initiating TKI treatment to the earliest sign of disease progression or death. Disease progression was determined using the Response Evaluation Criteria in Solid Tumors [40] in terms of complete response, partial response, stable disease, and progressive disease. The evaluation was performed via CT every three months as a routine clinical procedure as per the regulations of the National Health Insurance in Taiwan or other imaging methods (e.g., chest radiography, brain magnetic resonance imaging, bone scan, or positron emission tomography-CT) as needed during EGFR-TKI treatment. Both the physician and radiologist participated in the discussion of the disease progression to develop a proper treatment plan for the patients.

### Statistical analysis

Data were analyzed using SPSS for Windows, version 17.0 (Chicago, IL, USA). Continuous variables were reported as means ± standard deviations and were compared using two-tailed Student's *t* tests. Categorical variables were reported as the numbers of patients and percentages. Differences between categorical variables were evaluated using Chi-square or Fisher's exact test. The Kaplan-Meier method was used to generate PFS curves. The log-rank test was used to compare survival curves among patient groups. We used Cox proportional hazards models to adjust variations in the baseline characteristics. All statistical tests were two sided; a *p* value < 0.05 was considered significant.
